# Transcriptome Profiling Reveals Stage-Specific Production and Requirement of Flagella during Biofilm Development in *Bordetella bronchiseptica*

**DOI:** 10.1371/journal.pone.0049166

**Published:** 2012-11-12

**Authors:** Tracy L. Nicholson, Matt S. Conover, Rajendar Deora

**Affiliations:** 1 National Animal Disease Center, Agricultural Research Service, USDA, Ames, Iowa, United States of America; 2 Program in Molecular Genetics, Wake Forest University Health Sciences, Winston-Salem, North Carolina, United States of America; 3 Department of Microbiology and Immunology, Wake Forest University Health Sciences, Winston-Salem, North Carolina, United States of America; East Carolina University School of Medicine, United States of America

## Abstract

We have used microarray analysis to study the transcriptome of the bacterial pathogen *Bordetella bronchiseptica* over the course of five time points representing distinct stages of biofilm development. The results suggest that *B. bronchiseptica* undergoes a coordinately regulated gene expression program similar to a bacterial developmental process. Expression and subsequent production of the genes encoding flagella, a classical Bvg^−^ phase phenotype, occurs and is under tight regulatory control during *B. bronchiseptica* biofilm development. Using mutational analysis, we demonstrate that flagella production at the appropriate stage of biofilm development, i.e. production early subsequently followed by repression, is required for robust biofilm formation and maturation. We also demonstrate that flagella are necessary and enhance the initial cell-surface interactions, thereby providing mechanistic information on the initial stages of biofilm development for *B. bronchiseptica*. Biofilm formation by *B. bronchiseptica* involves the production of both Bvg-activated and Bvg-repressed factors followed by the repression of factors that inhibit formation of mature biofilms.

## Introduction

*Bordetella bronchiseptica* is a gram negative bacterial pathogen with a broad host range that naturally infects a wide variety of farm and companion animals [1], [2], [3]. It is the etiological agent or a co-contributor to a number of veterinary syndromes such as kennel cough in dogs, atrophic rhinitis (AR) and pneumonia in pigs and bronchopneumonia in guinea pigs, rabbits, horses, rats, mice, cats and nonhuman primates. *B. bronchiseptica* is also being increasingly isolated from humans mainly from immunocompromised patients. In many of these cases, the infections are caused by exposure to pets with *B. bronchiseptica* [4], [5].

A hallmark of *B. bronchiseptica* infections is long-term to life-long asymptomatic carriage. Despite vaccination, these bacteria continue to circulate and persist in animals. *B. bronchiseptica* is frequently isolated from the nasal cavities of vaccinated animals suggesting that vaccines fail to protect animals from infection [1], [2]. Additionally, *B. bronchiseptica* is capable of establishing a chronic and asymptomatic infection and can be harvested from the nasal cavities of rats, mice, and swine for extended periods [6], [7], [8], [9], [10]. We have isolated *B. bronchiseptica* from the rat nasopharynx even after 85 days of inoculation (our unpublished results) and the nasal cavity of laboratory mice remains colonized by *B. bronchiseptica* for the life of the animal [11].

A principal impediment towards the development of improved vaccines and interventions for *Bordetella* Spp. is a gap in our understanding of the mechanisms that contribute to persistence or the carrier state. A convincing and frequently proposed hypothesis to explain the survival and continued persistence of bacterial pathogens is that these organisms exist as biofilms. Recent studies have supported this hypothesis for members of the *Bordetella* genus by demonstrating that both *B. pertussis* and *B. bronchiseptica* are capable of forming biofilms on abiotic surfaces [12], [13], [14], [15], [16] and in the mouse respiratory tract [12], [17], [18], [19]. Biofilms are defined as a community of microorganisms enveloped in a hydrated extracellular polymeric matrix that adheres to the interface of a liquid and a surface. Numerous studies have documented increased resistance of biofilms to antibiotic treatments and the components of the host immune system [20], [21], [22], [23], [24]. Thus, bacterial biofilms are increasingly recognized as important contributors to chronic or persistent infections [25], [26].

We and others have previously shown that biofilm formation in *Bordetella* is regulated by BvgAS, a two-component sensory transduction system [13], [15]. This locus comprises a sensor kinase protein, BvgS, and a DNA-binding response-regulator protein, BvgA. In response to environmental cues, BvgAS controls the expression of a spectrum of phenotypic phases transitioning between a virulent (Bvg^+^) phase and a non-virulent (Bvg^−^ phase), a process referred to as phenotypic modulation. During the virulent Bvg^+^ phase, the BvgAS system is fully active and many of the known virulence factors are expressed [27]. Conversely, BvgAS is inactive during the Bvg^−^ phase, resulting in the maximal expression of motility loci, virulence-repressed genes (*vrg*), and genes required for the production of urease [28], [29], [30]. Previous studies have demonstrated that the Bvg^+^ phase is required for respiratory tract colonization [6], [31], [32], [33], [34], while the Bvg^−^ phase of *B. bronchiseptica* likely promotes survival outside of the mammalian host [31], [32].

Biofilm formation initiates with planktonic bacteria attaching to a surface leading to the formation of a monolayer, followed sometimes by formation of clusters and microcolonies and subsequent development of differentiated structures in which individual bacteria as well as the entire community are surrounded by an extracellular matrix [35], [36], [37]. For *B. bronchiseptica*, we have shown that the Bvg-mediated control of biofilm development is exerted subsequent to the initial attachment of the bacterial cells to a surface. We have also shown that the *B. bronchiseptica* Bps polysaccharide, which is not regulated by BvgAS, promotes biofilm formation at steps post-attachment, specifically in the development of three-dimensional structures [14], [18], [38]. Thus, for *B. bronchiseptica*, while some information has been obtained about the factors and the regulatory mechanisms that mediate later stages of biofilm development, nothing is known regarding early biofilm stages.

Biofilm cells differ from their planktonic cell counterparts in the genes and proteins they express, resulting in distinct physiological states and phenotypes [24], [39], [40]. Microscopic and genetic analysis has indicated that biofilm formation occurs in a sequential stage-specific and coordinated manner similar to microbial development, such as sporulation by *Bacillus* species and swarmer-to-stalk cell transition in *Caulobacter cresentus* [41]. The purpose of this study was to gain insights into the gene-expression profile and the transcriptional control operative in the biofilm associated *B. bronchiseptica* as a basis for understanding the contribution of individual genes in the various stages of biofilm development. Our analysis led to the surprising discovery that the expression of the genes encoding flagella, a classical Bvg^−^ phase phenotype, occurs early and is under tight regulatory control during *B. bronchiseptica* biofilm development. We have obtained convincing evidence that flagella are critical during the initial stages of biofilm formation. Our data suggest that the regulatory mechanism coordinating biofilm development in *B. bronchiseptica* results in the production of a classical Bvg^−^ phase phenotype under Bvg^+^ phase conditions.

## Materials and Methods

### Bacterial Strains and Plasmids

The strains and plasmids utilized in this study are listed in Table 1. *Bordetella* strains were grown in Stainer-Scholte (SS) broth [42] for both biofilm and planktonic cultures. The bacteria were maintained on Bordet Gengou (BG) agar (Difco, Sparks, MD) supplemented with 7.5% defibrinated sheep blood to determine hemolysis and colony morphology. The green fluorescent protein (GFP) expression vector pTac-GFP [14] was mobilized in strain RB50, Rev1*ΔflaA*, WT*ΔfrlAB*, or Bvg^−^ *ΔflaA* by triparental mating as described previously [43], and exconjugates were selected on BG agar containing 50 µg/mL chloramphenicol and 50 µg/mL streptomycin. Randomly picked colonies containing pTac-GFP were grown in SS broth containing 50 µg/mL chloramphenicol and were analyzed for GFP expression utilizing a Nikon Eclipse TE300 inverted microscope. One of the GFP-expressing clones corresponding to each of the strains was chosen for experimental analysis. Comparison of the GFP-expressing strains with the respective parental strains not containing the pTac-GFP plasmid revealed no differences in growth in batch cultures or colony morphology on BG agar containing blood. Strains containing the pTac-GFP plasmid were grown in the presence 50 µg/mL chloramphenicol.

**Table 1:** Strains and plasmids used in this study.

Strain or plasmid	Description	Reference or source
Strains		
RB50	Wild type *B. bronchiseptica* isolate	[31]
RB54	Bvg^−^ phase-locked; *ΔbvgS* derivative of RB50	[31]
Rev1	RB50 frl^r^; Replacement of the *frlAB* with the *fhaB* promoter resulting inexpression of flagella in the Bvg^+^ phase;	[6]
Rev1*ΔflaA*	nonflagellated; *ΔflaA* derivative of Rev1	[6]
Bvg^−^ *ΔflaA*	nonflagellated; *ΔflaA* derivative of RB54	[59]
WT*ΔfrlAB*	nonmotile; *ΔfrlAB* derivative of RB50	[6]
Plasmids		
pTAC-GFP	CM^r^ derivative of pBBR1-GFP	[14]
pKK233-2	hybrid trp/lac (*tac*) promoter	[60]
pBBR1-GFP	promoterless GFP, CAT vector	[61]

### RNA Isolation

RNA was extracted using a previously described method [44]. Briefly, single colony was inoculated in SS broth containing 40 µg/mL streptomycin at 37°C with shaking. Bacteria were then subcultured at a starting optical density at 600 nm (OD_600_) of 0.05–0.1 into 150×15 mm petri dishes (Fisher Scientific, Pittsburgh, PA) and incubated at 37°C for the indicated amount of time. A 10 mL aliquot of cells was removed for preparation of planktonic RNA, centrifuged, and then lysed with 1 ml RLT (Qiagen, Valencia, CA). The remaining media was removed, attached cells were washed three times with cold PBS and the remaining cells were lysed *in situ* with 1 mL of RLT buffer (Qiagen, Valencia, CA) and then isolated using the Qiagen RNeasy Kit (Qiagen, Valencia, CA).

### Preparation of Labeled cDNA and Microarray Analysis

A 2-color hybridization format was used for the microarray analysis. For each biological replicate, prepared RNA was first treated with DNase1 (Promega, Madison, WI) to remove genomic DNA and then RNA extracted from biofilm cells and used to generate Cy5-labeled cDNA and RNA extracted from planktonic cells was used to generate Cy3-labeled cDNA. Additionally, dye-swap experiments were performed analogously, in which the fluorescent labels were exchanged to ensure that uneven incorporation did not confound our results. Fluorescently-labeled cDNA copies of the total RNA pool were prepared and the two differentially labeled reactions to be compared were then mixed, followed by buffer exchange, purification, and concentration as described [45], [46], [47], [48]. The two differentially labeled reactions to be compared were combined and hybridized to a *B. bronchiseptica* strain RB50 specific long-oligonucleotide microarray [48]. Slides were then scanned using a GenePix 4000B microarray scanner and analyzed with GenePix Pro software (Axon Instruments, Union City, CA). Spots were assessed visually to identify those of low quality and arrays were normalized so that the median of ratios across each array was equal to 1.0. Spots of low quality were identified and were filtered out prior to analysis. Ratio data from the biological replicates were compiled and normalized based on the total Cy3% intensity and Cy5% intensity to eliminate slide to slide variation. Gene expression data were then normalized to 16 S rRNA. The statistical significance of the gene expression changes observed was assessed by using the significant analysis of microarrays (SAM) program [49]. A one-class unpaired SAM analysis using a false discovery rate of 0.001% was performed. Hierarchical clustering of microarray data using Pearson Correlation metrics and Average Linkage clustering was performed using MeV software from TIGR [50]. All microarray data are available in the supplementary text and have been deposited in ArrayExpress under accession number E-MEXP-3295.

### cDNA Preparation and Quantitative RT-PCR

Prepared RNA was first treated with DNase1 (Promega, Madison, WI) to remove genomic DNA and then reverse transcribed using Super Script III (Invitrogen, Carlsbad, CA). A mixture of 10 mM dNTPs (Invitrogen, Carlsbad, CA), 300 ng/µL random primers (Invitrogen, Carlsbad, CA), and 20 µL of RNA was incubated at 65°C for 5 min. After incubation first strand buffer, 10 mM DTT, and RNase OUT were added and incubated at room temperature for 10 minutes. Super Script III was subsequently added and incubated at 42°C for 50 minutes followed by inactivation at 70°C for 15 min. Mock reactions were preformed in parallel, without the addition of reverse transcriptase, to check for genomic contaminants. cDNA was then stored at −20°C until needed. Real time reactions were carried out in a final volume of 25 µL containing 2 µL of cDNA, Taqman mix (Applied Biosystems, Foster City, CA), and the primer pair. Primers were designed using Primer Express (Applied Biosystems, Foster City, CA). The probe was labeled using d-Fam as the quencher and d-Tam as the fluorophore. The PCR parameters were an initial cycle of 50°C for 2 minutes followed by 95°C for 10 minutes, followed by 40 cycles of 95°C for 15 seconds and 60°C for 1 minute. Calculations for comparison between samples were performed using genomic standard curve analysis with *rpoD* being used as the standardization control. Results were analyzed for significance using the Student’s *t* test and a *P* value less than 0.005 was considered significant.

### Microtiter Plate Assay of *Bordetella* Biofilm Formation

Biofilm development was examined using a microtiter dish assay as previously described [14]. *Bordetella* strains were inoculated at an OD_600_ of 0.05 into 100 µL of SS broth in a 96-well polyvinylchloride (PVC) microtiter plate. The plates were sealed with tape and then incubated at 37°C. At indicated time points the media and planktonic cells were removed and the plates were vigorously washed to remove loosely adherent cells. The remaining cells were stained with 0.1% crystal violet (CV) and incubated at room temperature for 30 minutes and then washed. CV staining the cells was solubilized with 200 µL of 95% ethanol. The solubilized CV was quantitated by transferring 125 µL to a new microtiter dish and determining the OD_540_. Results were analyzed for significance using the Student’s *t* test and a *P* value less than 0.05 was considered significant.

### Confocal Scanning Laser Microscopy

Mid log phase (OD_600_∼0.7–1.0) grown cultures of *B. bronchiseptica* strains carrying the pTac-GFP construct were inoculated in two chambered coverslips and cultured in SS broth with chloramphenicol. 12 mm glass coverslips were partially submerged in the culture and allowed to incubate for 48 hours at 37°C under static conditions. Biofilms formed at the air liquid interface of the coverslips were gently rinsed with PBS to remove any unattached bacteria and mounted with ProLong gold antifade reagent (Invitrogen, Carlsbad, CA), followed by visualization using a Zeiss LSM 510 confocal scanning laser microscope.

### Scanning Electron Microscopy

*Bordetella* strains were cultured statically on glass coverslips vertically submerged in SS broth, resulting in an air-liquid interface. At the indicated time points, the coverslips were removed, washed with sterile water and fixed using 2.5% glutaraldehyde for 1 hour. Samples were then processed for scanning electron microscopy as previously described [15], [18].

## Results

### *B. bronchiseptica* Exhibits a Coordinately Regulated Gene Expression Program During Biofilm Formation Similar to a Developmental Process

Previously, we have demonstrated that biofilm development in *B. bronchiseptica* is a microscopically observable sequential process that can be separated into distinct stages [14], [15]. Based on these data, we hypothesized that *B. bronchiseptica* undergoes a series of adaptive changes that allow it to successfully transition into sessile growth. A corollary of this hypothesis is that *B. bronchiseptica* cells within each biofilm stage exhibit gene expression patterns distinct from bacteria grown planktonically. To identify global stage-specific gene sets unique to biofilm formation for *B. bronchiseptica*, whole-genome transcriptome analysis was used to measure mRNA abundance under biofilm and planktonic growth conditions after 6, 12, 24, 36, and 48 hours of growth. A previously utilized static model of bacterial biofilm growth in petri dishes was used for these analyses [38], [44]. As shown in Figure S1, *B. bronchiseptica* cells adhered to the plastic surface were observed by SEM at 6 hours. At 12 hours, more cells adhered to the plastic surface; however these sessile cells lacked any apparent structural organization. By 24 hours, cell clusters separated by individual cells were apparent and by 48 hours cells were present mainly as mature macrocolonies encased in an opaque white matrix-like material (Figure S1). Therefore, this static biofilm system replicates the developmental stages of bacterial biofilms and allows transcriptome analysis during planktonic and biofilm growth under identical growth conditions.

**Figure 1:**
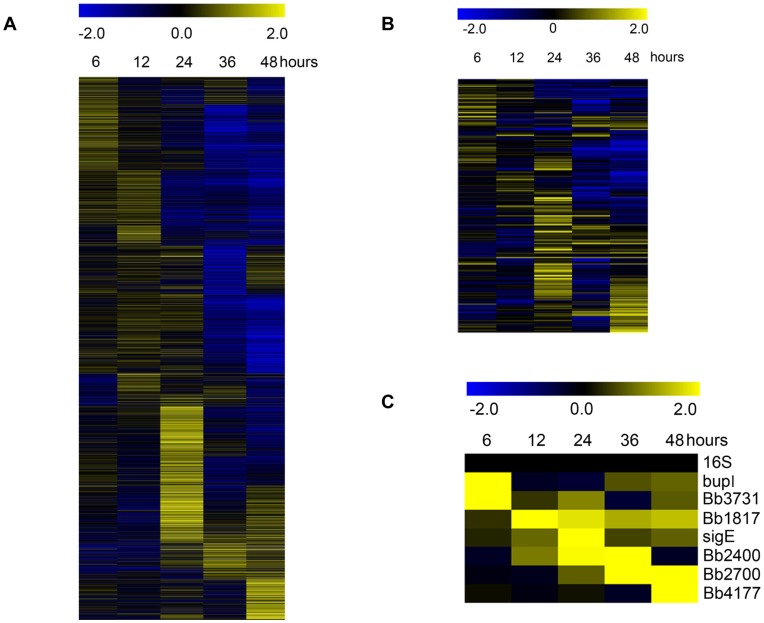
Hierarchical clustering of the transcriptional response of *B. bronchiseptica* strain RB50 throughout biofilm development identified by comparing cDNA from planktonic cells to biofilm cells at 6, 12, 24, 36, and 48 hours of growth. A) Expression profiles representing global transcriptional changes B) Expression profiles of annotated *B. bronchiseptica* transcription factors. C) Expression profiles of annotated *B. bronchiseptica* transcription factors maximally expressed under biofilm growth conditions at 6, 12, 24, 36, and 48 hours. Data are mean centered for each array element and averaged from three biological replicates. All expression profiles of genes are in row and are represented using the color scale at top. Yellow, indicates increased expression in biofilm cells; blue, decreased gene expression in biofilm cells; black, no significant change in gene expression.

More than 33% of the *B. bronchiseptica* genes exhibited statistically significant transcriptional activation or repression during biofilm growth. A self-organizing map technique was applied to the biofilm regulated expression profiles to identify gene sets with similar expression patterns. As shown in Figure 1A, this clustering map reveals a periodicity or a cascade of continuous expression lacking clear boundaries or sharp transitions and demonstrates an orderly timing of gene expression extremely similar to global gene expression profiles occurring during bacterial development (Table S1) [41], [51], [52].

Gene expression occurring throughout bacterial development is governed by the production of transcription factors at the appropriate stages [51]. Therefore, the same self-organizing map technique was applied to the annotated *B. bronchiseptica* transcription factors revealing a parallel temporally regulated expression pattern as the global *B. bronchiseptica* gene expression profiles (Figure 1B and Table S1). To highlight this tight temporal expression pattern, transcription factors used in Figure 1B were trimmed to contain only those present in the greatest abundances at each point time (Figure 1C). Specifically, *bupI* Bb4742 and Bb3731, were found to be maximally expressed at 6 hours (Figure 1C). Bb1817 was found to be maximally expressed at 12 hours, while *sigE* Bb3752 was found to be maximally expressed at 24 hours (Figure 1C). Bb2400 was found to be maximally expressed at 24 hours and 36 hours, and Bb2700 was found to be maximally expressed at 36 hours and 48 hours (Figure 1C). Lastly, Bb4177 was found to be maximally expressed at 48 hours (Figure 1C). This rigid expression pattern suggests that specific transcription factors or regulators are needed at distinct stages or times during biofilm development. A complete list of gene expression measurements during biofilm growth can be found in Table S1. Overall, the transcriptional profiles for both global gene expression and transcription factor-specific gene expression suggest that *B. bronchiseptica* undergoes a coordinately regulated gene expression program similar to a bacterial developmental process.

### Bvg^−^Phase Phenotype is Favored During Initial Stages of Biofilm Formation

**Figure 2:**
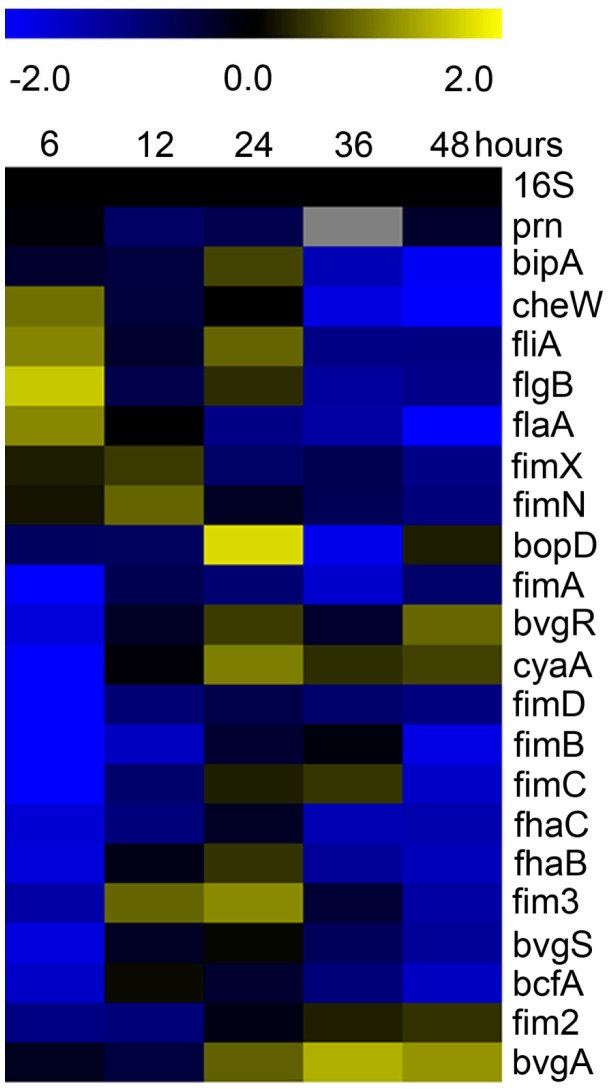
Hierarchical clustering of the transcriptional response of BvgAS-regulated genes throughout biofilm development identified by comparing cDNA from planktonic cells to biofilm cells at 6, 12, 24, 36, and 48 hours of growth. Expression profiles representing transcriptional changes of genes regulated by BvgAS, along with 16S. Data are mean centered for each array element and averaged from three biological replicates. All expression profiles of genes are in rows and are represented using the color scale at top with gray indicating missing data. Yellow, indicates increased expression in biofilm cells; blue, decreased gene expression in biofilm cells; black, no significant change in gene expression.

We and others have previously demonstrated that BvgAS is required for *B. bronchiseptica* biofilm development [13], [15]. However, gene expression occurring at distinct stages of biofilm development has not been investigated for many BvgAS-regulated factors. To address this, we applied a self-organizing map technique to genes encoding classical BvgAS-regulated factors, such as adhesins, toxins, and those contributing to flagella synthesis and motility (Figure 2). This analysis revealed that many of the positively regulated BvgAS genes were repressed at 6 hours, while many negatively regulated BvgAS genes were found to be induced. Specifically, genes involved in motility (*cheW*, *fliA*, *flgB*, *flaA*) were upregulated, while *bvgR* and the genes positively regulated by BvgAS (*bopD*, *fimA*, *cyaA*, *fimD*, *fimB*, *fimC*, *fhaC*, *fhaB*, *fim3*, *bvgS*, *bcfA*, and *fim2*) were repressed under biofilm growth conditions at 6 hours (Figure 2). Of the known Bvg-activated genes, only *prn*, *bipA* and *bvgA* were found to be expressed at similar levels under biofilm and planktonic growth conditions at 6 hours (Figure 2). The gene expression profiles of these classical BvgAS-regulated factors suggest that a Bvg^−^ phase phenotype is favored during early or initial stages of biofilm formation.

### The BvgAS-repressed Gene *flaA* is Differentially Expressed During *B. bronchiseptica* Biofilm Development

Perhaps the best characterized phenotype of the Bvg^−^ phase is motility. The expression of genes involved in the synthesis of flagella is tightly regulated through a complex hierarchy requiring the presence of the regulatory proteins, FrlA and FrlB, and the production of the flagellin monomer, FlaA. BvgA negatively regulates the production of flagella and subsequent motility in *B. bronchiseptica* by repressing the expression of *frlA* and *frlB* [6], [28], [29]. *frlA and frlB* encode activators of flagellar genes and have been hypothesized to regulate *flaA* expression through a *fliA* analogue in *Bordetella* [6].

**Figure 3:**
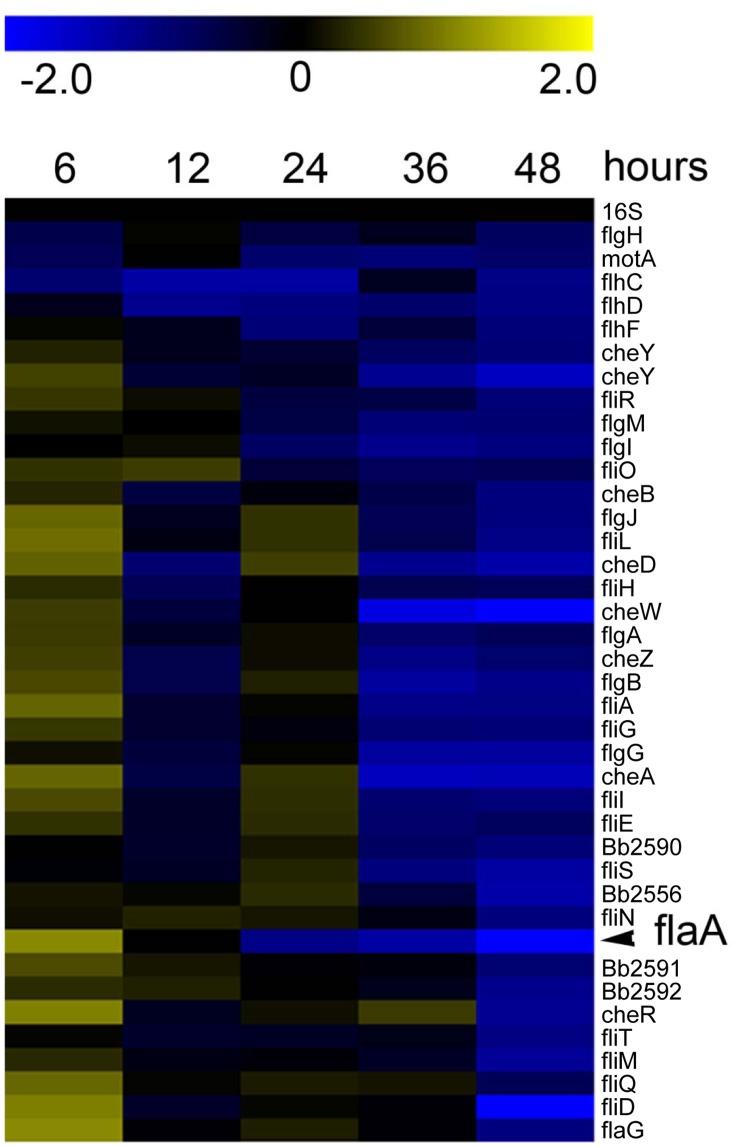
Hierarchical clustering of the transcriptional response of genes located within the motility locus throughout biofilm development identified by comparing cDNA from planktonic cells to biofilm cells at 6, 12, 24, 36, and 48 hours of growth. Expression profiles representing transcriptional changes of genes located within the motility locus, along with 16S. Data are mean centered for each array element and averaged from three biological replicates. All expression profiles of genes are in row and are represented using the color scale at top. Yellow, indicates increased expression in biofilm cells; blue, decreased gene expression in biofilm cells; black, no significant change in gene expression. The flagella structural gene *flaA* is indicated.

As shown in Figure 3A, we found that the majority of genes located within the motility locus exhibited transcriptional changes throughout *B. bronchiseptica* biofilm development. In general, these transcriptional changes were inversely related to later time points such that the expression of many of these genes were upregulated or increased early at 6 hours and then decreased late at 48 hours (Figure 3). The flagella structural gene *flaA* is marked as an example of this inverse or differential gene expression pattern (Figure 3). However, *frlA* and *frlB* were observed to be similarly expressed in both planktonic and biofilm conditions at 6 hours followed by a subsequent repression at 48 hours (Table S1).

**Figure 4:**
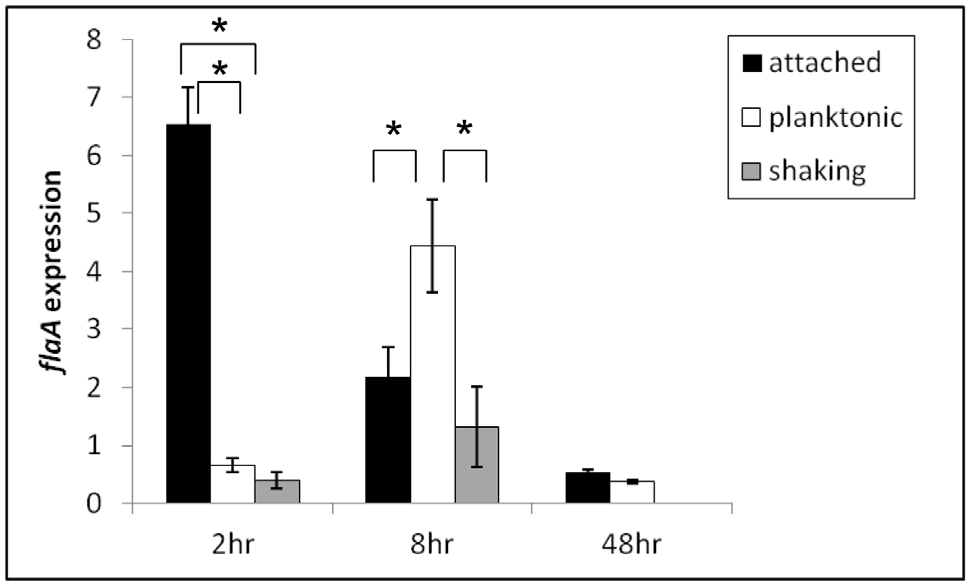
Differential expression of *flaA* during biofilm development. Gene expression of the flagella structural gene *flaA* as determined by quantitative RT-PCR in attached, planktonic, or cells grown in shaking culture conditions at 2, 8, and 48 hours of growth. Gene expression standardized to *rpoD* is plotted along the y-axis and attached, planktonic, and shaking culture conditions are shown along the x-axis. Data shown are averages obtained from triplicate cultures. The error bars represent +/− the standard deviation. Asterisks represent a p value less than 0.005.

To independently verify the gene expression pattern of *flaA*, encoding the flagellin monomer, quantitative RT-PCR was performed using RNA isolated from cells adhered to polystyrene plates during early (2 and 8 hours) and late (48 hours) stages of biofilm development. We additionally included RNA isolated from cells grown in static planktonic and from cells grown in shaking culture conditions. *flaA* expression was highest in bacteria attached to the plate at 2 hours (Figure 4). At this time point, the expression of *flaA* in either the planktonic or the shaking cultures was comparatively lower (Figure 4). The expression of *flaA* is repressed in attached cells at 8 hours followed by very low expression at 48 hours (Figure 4). In contrast, *flaA* expression increases to reach maximum levels at 8 hours in cells grown in static planktonic and shaking culture conditions (Figure 4). Similar to that observed for the sessile cells, cells grown in static planktonic and shaking culture conditions for 48 hours, expressed *flaA* at comparatively lower levels (Figure 4). By 48 hours, there are viable bacteria in all the three populations as determined by plating on BG agar plates and by the observation that *rpoD* transcript levels are still detectable. These data confirm the results from the microarray analysis by revealing the differential expression pattern of *flaA* during *B. bronchiseptica* biofilm development. Overall, the pattern of *flaA* expression indicates that surface-adherent bacteria preferentially express flagella at early times and repress the expression at later stages of biofilm formation.

### Flagella are Necessary for Initial Surface Contact but are not Necessary at the Later Stages of Biofilm Development

**Figure 5:**
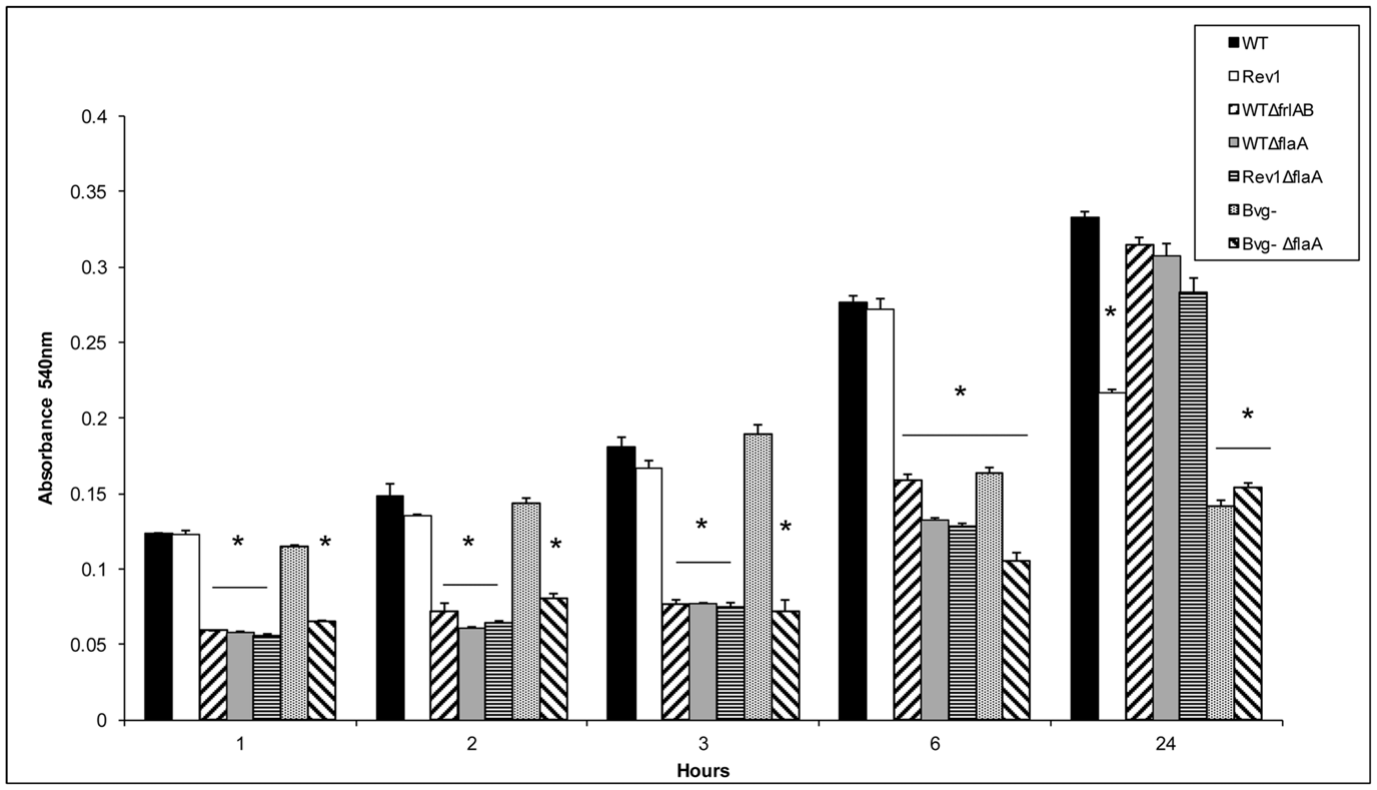
Flagella are necessary for initial surface contact but deleterious for biofilm maturation. Kinetics of biofilm development for the different strains was analyzed by the microtiter assay. Optical densities (OD_540_) of solubilized crystal violet from surface associated cells are plotted along the y-axis and time in hours, is shown along the x-axis. Data shown are averages obtained from at least 6 wells each time from 2 independent experiments. The error bars represent +/− the standard error. Asterisks represent a p value less than 0.05.

The upregulation of genes involved in motility and the flagellar synthesis, including the flagellin monomer *flaA*, at early time points of biofilm development suggested that flagella serve a critical role in *Bordetella* biofilm formation. We therefore hypothesized that flagella are necessary for establishing the initial interactions with the surface. To examine the role of flagella in initial surface contact, we utilized mutants containing deletions in either the *frlAB* or the *flaA* genes (Table 1). Biofilm formation was quantified by staining the cells attached to a polystyrene plate with crystal violet over a time course of 1, 2, 3 and 6 hours. Compared to the WT strain, which is capable of producing flagella, the strains containing deletions in either *frlAB* or *flaA* (WT*ΔfrlAB*, WT*ΔflaA*) and therefore lacking the ability to produce flagella were defective in forming biofilms for all the early time points examined (Figure 5). These data thus demonstrate that flagella are necessary for establishing initial surface interactions by the WT strain.

In addition to examining biofilm formation at early time points, we also analyzed the biofilms formed by these strains at 24 hours. Strains containing deletions in either *frlAB* or *flaA* genes (WT*ΔfrlAB*, WT*ΔflaA*) were able to form biofilms as robust as the WT by 24 hours, suggesting that the presence of flagella is not absolutely required for continued biofilm formation at later time (Figure 5).

Previously, we have shown that while the BvgAS signal transduction system is required for efficient biofilm formation in *Bordetella*, it is not essential for initial surface interactions [15]. Specifically, we showed that there were no significant differences in the adherence of the WT and the Bvg^−^ phase-locked strains to the microtiter plates at the early time points (Figure 5 and [15]). We also found that the Bvg^+^ and the Bvg^i^ phase locked strains were also defective in biofilm formation at early time points compared to the WT strain [15]. The Bvg^+^ phase-locked strain encodes a constitutively active BvgS protein that is insensitive to modulators, whereas the Bvg^i^ phase-locked strain is locked in the intermediate phase [32]. Both the Bvg^+^ and the Bvg^i^ strains are non-motile and do not produce flagella, whereas the Bvg^−^ phase locked strain is motile and produces flagella. We hypothesized that flagella are responsible for the observed early biofilm forming ability of the Bvg^−^ phase-locked strain. Compared to the Bvg^−^ phase-locked strain, the Bvg^−^ *ΔflaA* strain formed less biofilms at 1, 2, 3 and 6 hours (Figure 5). These results suggest that flagella can contribute to the initial biofilm formation by *B. bronchiseptica* in the absence of Bvg-activated factors. At 6 and 24 hours, compared to the WT strain, the Bvg^−^ phase locked strain was defective in biofilm formation (Figure 5). This suggests that expression of flagella in the absence of Bvg-activated factors is not sufficient for continued formation of efficient biofilms.

### Repression of Flagella is Essential for Biofilm Formation

Based on the inverse regulation of flagellar and motility gene expression during biofilm development, we hypothesized that while initial expression of flagella is necessary for enhancing initial surface contact, continued expression of flagella in the WT strain would be deleterious for biofilm development. To begin addressing the requirement for repression of flagella during later stages of biofilm development, we utilized a strain (Rev1) that ectopically expresses flagella in the wild-type background. Rev1 was previously generated by replacing the Bvg-repressed *frlAB* promoter with the Bvg-activated *fhaB* promoter [6].

**Figure 6:**
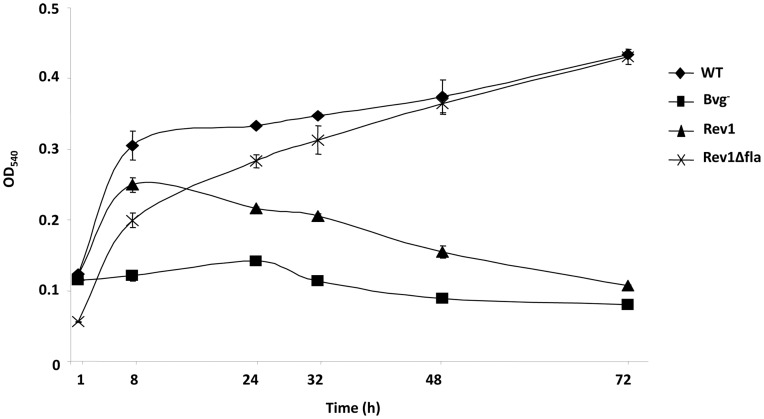
Flagella are deleterious for biofilm maturation. Kinetics of biofilm development for different strains was analyzed by the microtiter assay. Optical densities (OD540) are plotted along the y-axis and time in hours, is shown along the x-axis. Data shown are averages obtained from at least 6 wells each time from 2 independent experiments. The error bars represent +/− the standard error. All differences are statistically significant (p<0.05, student’s t test), except among the strains at 1 h time point and between the strains Rev1Δfla and WT at 48 and 72 hours.

To quantify biofilm formation, cells attached to the polystyrene plate were stained with crystal violet over a time course of 1, 2, 3, 6, 8, 24, 32, 48, and 72 hours. Consistent with the requirement of flagella at early stages of biofilm formation, the biofilms formed by the Rev1 strain was similar to that of the WT strain for up to 6 hours (Figures 5 and 6). At later time points, e.g. at 8 hours or later, the Rev1 strain formed decreased biofilms relative to the WT strain until reaching that of the Bvg^−^ phase-locked strain by 72 hours (Figure 6).

In the strain Rev1, the *frlAB* locus is ectopically expressed from the *fhaB* promoter. Since *frlAB* encodes transcriptional regulatory proteins, activation of this locus may lead to the expression of yet unidentified *frlAB*-regulated motility-independent genes, which may influence biofilm development. In order to address this caveat and to demonstrate that the ectopic expression of flagella is sufficient for the biofilm defect associated with the Rev1 strain, the Rev1 strain containing a deletion of the *flaA* gene (Rev1*ΔflaA*) (Table 1) was utilized. At early stages of biofilm formation, up to 8 hours, the adherence capacity of the Rev1*Δfla* strain, lacking the ability to produce flagella, was lower than that of the WT strain and the Rev1 strain (Figures 5 and 6). However, after 8 hours, the Rev1*Δfla* strain started to display increased biofilm formation compared to that formed by the Rev1 strain. At 48 and 72 hours, both the WT and the Rev1*ΔflaA* strain formed biofilms to similar levels (Figure 6). Taken together, these data demonstrate that ectopic expression of flagella is detrimental for continued biofilm formation at later time points.

### Microscopic Analysis of Biofilms Formed by the WT and Different Isogenic Mutants

**Figure 7:**
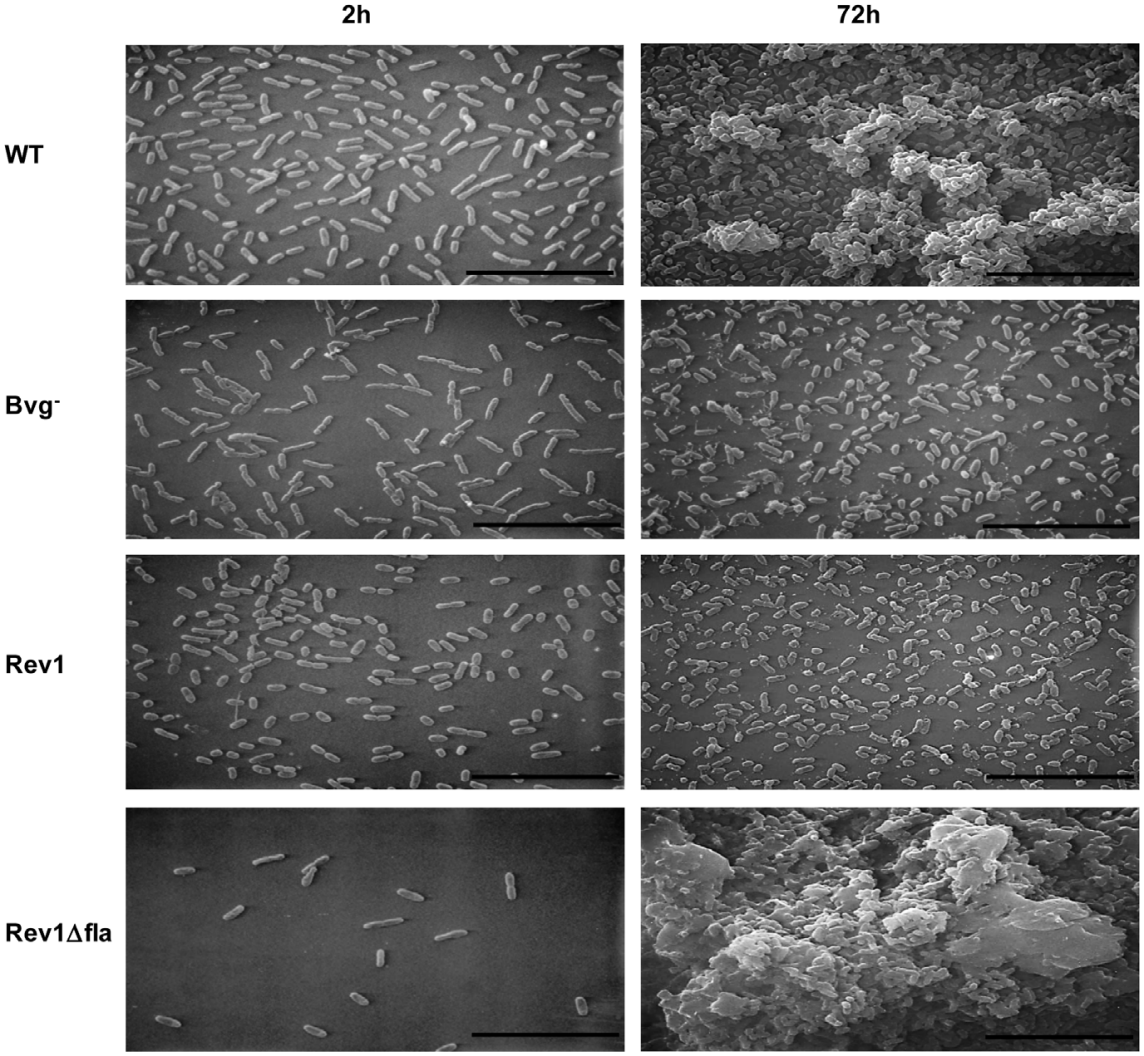
Scanning electron microscopy (SEM) of biofilms formed at the air-liquid interface. Different strains were grown on glass cover slips for 2 hours (left panels) and 72 hours (right panels) followed by processing for SEM as described in the Materials and Methods. The scale bar represents 10 µm.

To further define the role that flagella play in *B. bronchiseptica* biofilm development, we visualized architectural features of biofilms by scanning electron microscopy (SEM). Glass coverslips were vertically submerged in a bacterial suspension creating an air-liquid interface that was visualized using SEM. After 2 hours of incubation, only strains capable of producing flagella, WT, Bvg^−^, and Rev1 were found to adhere in significant numbers to the surface (Figure 7). Comparatively, the Rev1Δ*flaA* strain lacking the ability to produce flagella adhered sporadically to the surface after 2 hours with a substantial decrease in the number of cells adhering relative to the WT strain and the isogenic parent, Rev1 (Figure 7). At later stages of biofilm development i.e. at 72 hours, strains incapable of flagella repression, Rev1 and Bvg^−^, were unable to cluster together and form three dimensional structures. In contrast, the WT and the Rev1*Δfla* strains formed three dimensional structures encased in a matrix like material (Figure 7). These data demonstrate that flagella are needed to initiate contact with a surface. However, this defect can be compensated at later time points in strains that can repress or lack flagella production. The similar biofilm forming ability of the Rev1 and the WT strains further demonstrates that the potential *frlAB*-regulated motility-independent genes are not responsible for the biofilm defect exhibited by the Rev1 strain. Thus, the inability of the Rev1 strain to form mature biofilms is due to the failure to repress flagella production during later stages of biofilm formation.

**Figure 8:**
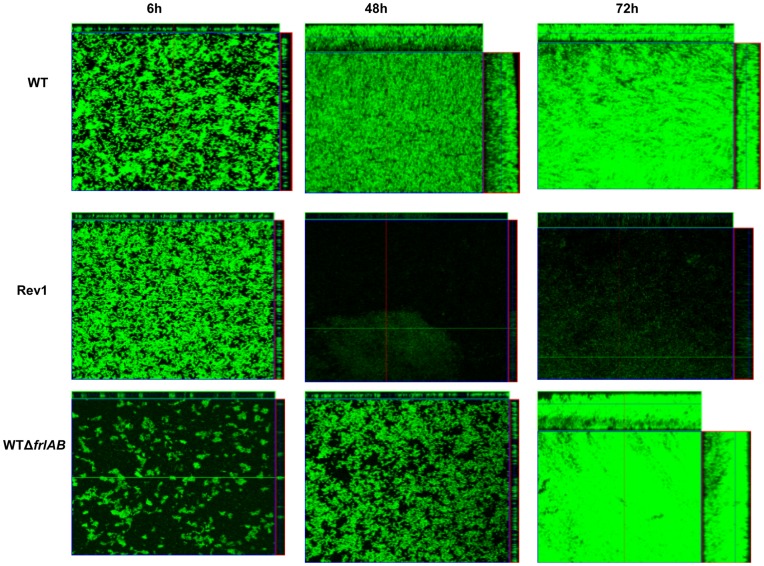
Confocal scanning laser micrographs (CSLM) of *B. bronchiseptica* biofilm development. CSLM of biofilms formed at the air-liquid interface of different strains grown on glass coverslips after 6 hours (left panels), 48 hours (middle panels), and 72 hours (right panels). The cells were tagged with GFP and thus are green. For each micrograph, the middle panel represents the *x–y* plane, and the adjacent top and side panels represent the *x–z* and *y–z* planes, respectively.

To further examine the contribution of flagella on impacting biofilm structure, we continued this experiment with GFP-expressing derivatives of the WT and the mutant strains. After 6 hours of incubation, the WT strain attached and covered a large area of the coverslip, which appeared to be completely occupied by 48 hours (Figure 8). By 72 hours, the entire coverslip had been extensively covered by the WT strain, resulting in the visualization of a thick layer of cells at the air-liquid interface (Figure 8). In contrast, the WTΔ*frlAB* strain was defective in surface-adherence at 6 hours as demonstrated with large areas of the coverslips remaining devoid of the bacterial cells. At 72 hours, the WTΔ*frlAB* strain had recovered from the initial defect in surface attachment and colonized the glass cover slip similar to the WT strain (Figure 8). Consistent with above results, while the Rev1 strain did not appear to be defective in attaching to the coverslip at 6 hours, the majority of the coverslip was essentially devoid of bacterial cells at 48 and 72 hours (Figure 8). These data confirm that the inability to repress flagella is detrimental for continued biofilm formation at later time points.

## Discussion

Biofilm formation has been proposed to be a process of microbial development similar to that observed in cell cycle-controlled swarmer-to-stalk cell transition in *Caulobacter crescentus*, sporulation in *Bacillus subtilis*, and fruiting-body formation by *Myxococcus xanthus* [41], [51]. This view is gaining acceptance as an increasing number of studies reveal alterations in bacterial cell physiology, along with the transcriptional program underlying these physiologies, which occur throughout the progression from planktonic to the biofilm state [36], [37], [44], [52], [53]. Despite the almost widespread acceptance of this model, it has been heavily criticized due to the lack of experimental evidence directly linking a stage-specific biofilm developmental phenotype with a temporal genetic program or mechanism. Recently, the existence of a regulatory program for stage-specific biofilm development in *Pseudomonas aeruginosa* has been demonstrated by Petrova and Sauer [52]. We have used microarray analysis to study the transcriptome of *B. bronchiseptica* over the course of five time points representing distinct stages of biofilm development (Figure S1 and [15]). Our data revealed prominent shifts in the global transcriptional program that occur at distinct stages of biofilm development. Annotated *B. bronchiseptica* transcription factors were additionally found to have a similar temporally regulated expression pattern as the global *B. bronchiseptica* gene expression profiles. This temporally regulated expression pattern suggests that specific transcription factors are needed at distinct stages or times during biofilm development providing experimental evidence linking stage-specific biofilm phenotypes to a sequential genetic mechanism. Overall, both the transcription factor and global gene expression profiles suggest that *B. bronchiseptica* undergoes a coordinately regulated gene expression program during biofilm development similar to a bacterial developmental process.

Global transcriptome analysis led to the surprising discovery that many of the *Bordetella* genes previously known to be positively regulated by the BvgAS signal transduction system were repressed during early stages of biofilm development, while many negatively regulated BvgAS genes were found to be induced. This finding was unexpected since biofilm formation and maturation requires BvgAS activation and well documented Bvg^+^ phase conditions, such as growth at 37°C and the absence of chemical modulators [15], [17], [18]. The observed gene expression pattern further suggested that a Bvg^−^ phase phenotype is favored during initial stages of biofilm formation. Microarray analysis additionally led to the discovery that expression of flagella occurs and is under tight regulatory control during *B. bronchiseptica* biofilm development such that the expression of many of the genes located within the motility locus were either upregulated early at 6 hours, including the flagellin monomer, *flaA*, or similarly expressed in both planktonic and biofilm conditions at 6 hours, such as *frlA* and *frlB*, followed by a subsequent repression late at 48 hours.

Using mutational analysis in a combination of several different assays, we confirmed the microarray data and demonstrated that *B. bronchiseptica* expresses flagella early during biofilm development and then subsequently represses the expression of flagella. *B. bronchiseptica* strains unable to produce flagella failed to bind to a surface at early stages of biofilm formation. However, this defect is ameliorated with time since strains that lack flagella production were able to form biofilms as robust as the WT strain by late biofilm stages. Together, the presented data demonstrate that flagella are needed to contact a surface; however, they are not required for biofilm maturation.

The expression of *B. bronchiseptica* flagellar synthesis genes are thought to be regulated through a complex hierarchy similar to the cascade of transcriptional events that occur in *E. coli* [54]; however there are many details of this regulatory hierarchy that remain undetermined. To summarize our current understanding, BvgAS lies at the top of the hierarch and negatively regulates the expression of *frlA* and *frlB* [6], [29]. FrlAB is required for the production of flagella and has been hypothesized to regulate *fliA*, which in turn upregulates *flaA*, encoding the flagellin monomer, required for the production of flagella [6], [28]. In other microorganisms, genes required for the synthesis of flagella have been found to be repressed in the biofilm cells leading to suggestions that flagella repression is a key step in biofilm development [35], [55], [56]. However, to our knowledge, the requirement to repress flagella production during biofilm development has not been directly demonstrated. Using a series of mutants that allowed us to alter the known mechanisms within the regulatory hierarchy for *B. bronchiseptica* flagella production, we demonstrated that flagella repression is absolutely required for formation of mature and structured biofilms.

Data presented in this study were obtained from a variety of *in vitro* model systems. As with all *in vitro* model systems, possible artifacts may include high starting bacterial density in nutrient rich broth, oxygen, and nutrient availability. These culture conditions can initially begin with a fully aerated culture subsequently developing an oxygen gradient from the surface to the bottom of the culture. Additionally, gradients within biofilms can often form and result in localized microenvironments. All of these possible environmental parameters are likely to affect gene expression. Also, bacterial products that are either required for or enhance adherence to abiotic surfaces may have a different contributing role in the development and maturation of biofilms formed in the environment or within a host.

In conclusion, it is clear from these studies that biofilm development in *Bordetella* is under the control of a complex regulatory circuitry requiring the coordinated stage-specific expression of multiple transcription factors. One outcome of this regulatory hierarchy is the transient expression of a Bvg^−^ phase phenotype under Bvg^+^ phase conditions. *B. bronchiseptica* is able to survive and grow in soil for extended periods, in a nutrient-limiting environment and at temperatures as low as 10°C [57]. The Bvg^−^ phase of *B. bronchiseptica* has been demonstrated to promote bacterial survival under conditions of nutrient deprivation [31], [57]. Thus, the expression of Bvg**-**associated traits such as flagella could lead to efficient biofilm formation in the environment and thus contribute to survival in environmental niches. There may also be a possible infectious benefit of the expression of flagella as well. It has previously been demonstrated that the Rev1 mutant colonized the nasal cavities of rats similarly to wild-type *B. bronchiseptica* strain, demonstrating that expression of flagella is not deleterious to nasal colonization [6]. The lower temperature (lower than 37°C) of the anterior portions of the upper respiratory tract may also allow expression of flagella by the WT strain during initial stages of infection. Additionally, purified flagella from *B. bronchiseptica* have been shown to adhere to epithelial cells [58]. Therefore, by initiating biofilm development during early infection, possibly through adherence, flagella may also promote survival within nasal cavities of hosts, thereby increasing transmission dynamics within host populations or between animals.

## Supporting Information

Figure S1 **Scanning Electron Microscopy (SEM) of biofilms formed on polystyrene Petri plates.** Logarithmic phase cultures inoculated into the Petri plate and grown at 37°C for the indicated time followed by processing for SEM as described in the Materials and Methods. The scale bar represents 10 µm.
(TIF)

Table S1 **Sheet 1.** Whole Genome Microarray data: **Fold-Change expression values during biofilm growth in** ***B. bronchiseptica*** **RB50.** DNA microarray analysis was used to measure mRNA levels present in *B. bronchiseptica* RB50 planktonic cells compared to biofilm cells at 6, 12, 24, 36, and 48 hours of growth. Differences in mRNA levels are listed as mean fold-changes ± standard deviation. Fold-changes were calculated by averaging the data from three biological replicates for 6, 12, and 24 hour time points and two biological replicates for 36 and 48 hour time points. The fluorescent labels were exchanged in dye-swap experiments performed on all biological replicates. Genes identified as significantly regulated by growth phase (“Negative” or “Positive”) were assessed by using the significant analysis of microarrays (SAM) program [49]. A one-class unpaired SAM analysis using a false discovery rate of 0.001% was preformed and the Score(d), q-value, and localdr(%) columns are given. **Sheet 2.** Trx regulator microarray data**: Fold-Change expression values of transcriptional regulators in** ***B. bronchiseptica*** **RB50 during biofilm growth.** DNA microarray analysis was used to measure mRNA levels present in *B. bronchiseptica* RB50 planktonic cells compared to biofilm cells at 6, 12, 24, 36, and 48 hours of growth. Differences in mRNA levels are listed as mean fold-changes ± standard deviation. Fold-changes were calculated by averaging the data from three biological replicates for 6, 12, and 24 hour time points and two biological replicates for 36 and 48 hour time points. The fluorescent labels were exchanged in dye-swap experiments performed on all biological replicates. Genes identified as significantly regulated by growth phase (“Negative” or “Positive”) were assessed by using the significant analysis of microarrays (SAM) program [49]. A one-class unpaired SAM analysis using a false discovery rate of 0.001% was preformed and the Score(d), q-value, and localdr(%) columns are given.
(XLSX)
