# Supplementary figures and images for: Transcriptome Profiling Reveals Stage-Specific Production and Requirement of Flagella during Biofilm Development in Bordetella bronchiseptica
Source: PLoS One. 2012 Nov 12;7(11):e49166. doi: 10.1371/journal.pone.0049166 (PMC3495763; doi:10.1371/journal.pone.0049166)

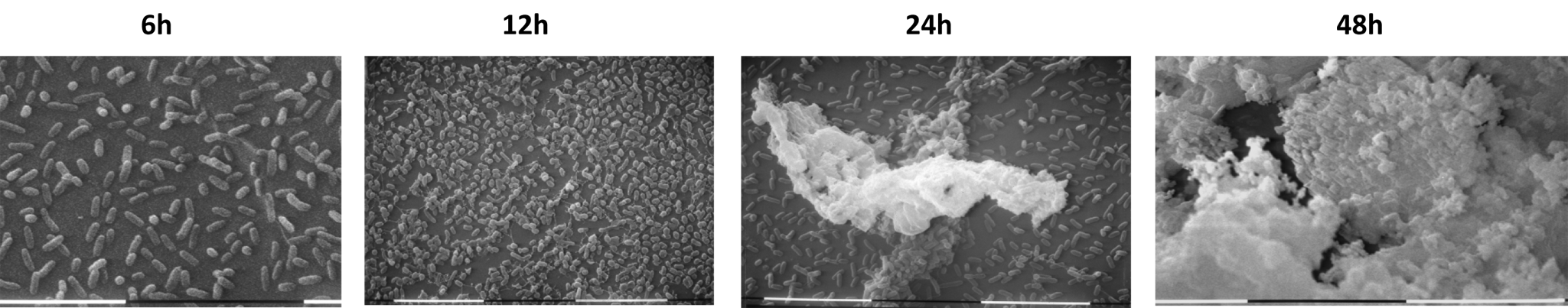

Supplement: Figure S1 — Scanning Electron Microscopy (SEM) of biofilms formed on polystyrene Petri plates. Logarithmic phase cultures inoculated into the Petri plate and grown at 37°C for the indicated time followed by processing for SEM as described in the Materials and Methods. The scale bar represents 10 µm. (TIF) [file pone.0049166.s001.tif]
